# Multivariate Characterization of Essential Oils for Their Antibacterial Activity Against *Escherichia coli*: A Data-Driven Interpretation of Experimental Results

**DOI:** 10.3390/molecules31020207

**Published:** 2026-01-07

**Authors:** Meta Kokalj Ladan, Marsela Supé Vide, Katja Schoss

**Affiliations:** Faculty of Pharmacy, University of Ljubljana, Aškerčeva 7, 1000 Ljubljana, Sloveniakatja.schoss@ffa.uni-lj.si (K.S.)

**Keywords:** essential oils, *Escherichia coli*, antimicrobial activity, GC–MS analysis, multivariate analysis, bioactive compounds

## Abstract

The growing problem of antimicrobial resistance emphasizes the urgent need for new and effective natural antimicrobial agents. This study assessed the antibacterial activity of twenty essential oils and one absolute against *Escherichia coli* and examined the relationship between their chemical composition and biological activity. The chemical profiles of the samples were determined using gas chromatography–mass spectrometry (GC–MS), and the resulting data were analysed using principal component analysis (PCA), discriminant analysis (DA), and partial least squares (PLS) methods to explore associations between composition and antibacterial activity. The results showed substantial variability among the tested essential oils, with those from *Thymus vulgaris*, *Aniba rosaeodora*, *Syzygium aromaticum*, *Pimenta dioica*, and the absolute of *Evernia prunastri* exhibiting the strongest activity. GC–MS analysis identified thymol, eugenol, and methyl atrarate as key bioactive constituents associated with strong antibacterial effects, while linalool, limonene, and α-terpineol were linked to moderate activity. Multivariate analyses provided further insight but were limited by data variability, highlighting compositional diversity rather than clear group separation. Overall, the findings demonstrate that essential oils are a promising source of natural antimicrobial agents and emphasise the importance of linking chemical composition with biological function to understand their potential therapeutic applications.

## 1. Introduction

The increasing frequency of antimicrobial-resistant microorganisms represents a major global health challenge and is now recognised as one of the most serious threats to human health [1,2]. A primary challenge in the pharmaceutical and healthcare sectors is multidrug resistance, which complicates the treatment of bacterial infections and has been further intensified by the overuse and inappropriate prescribing of antibiotics, as well as the impact of the COVID-19 pandemic [3,4]. Studies on antimicrobial resistance estimate that 4.95 million deaths in 2019 were associated with infections involving antimicrobial-resistant bacteria [5], highlighting the urgent need to discover new antimicrobial agents.

*Escherichia coli*, a Gram-negative, facultatively anaerobic bacterium, is one of the most extensively studied microorganisms and serves as an important model organism in microbiology and biotechnology [6]. While most strains are harmless commensals inhabiting the intestinal tract of humans and animals, certain pathogenic variants can cause serious infections, including urinary tract infections, neonatal meningitis, and gastroenteritis [7,8]. Due to its genetic adaptability and ability to acquire resistance genes, *E. coli* has become a frequent cause of multidrug-resistant infections. The increasing prevalence of resistant *E. coli* strains, including extended-spectrum β-lactamase (ESBL)-producing and carbapenem-resistant isolates, emphasises the need for alternative antimicrobial strategies [9,10].

The continuous rise in antimicrobial resistance has renewed scientific interest in natural products as potential sources of novel antimicrobial agents. Historically, plants, fungi, and microorganisms have formed the basis of drug discovery, with many modern drugs originating from natural sources [11]. Plant-derived compounds, in particular, are known for their structural diversity and bioactivity [12]. Their specialised metabolites, including alkaloids, flavonoids, terpenes, and phenolic compounds, play important ecological roles in plant defence mechanisms and have demonstrated promising antibacterial, antifungal, and antiviral effects [13,14].

Among plant-derived natural products, essential oils (EOs) have attracted increasing attention due to their broad-spectrum antimicrobial activity, availability, and versatility in pharmaceutical, cosmetic, and food industry applications [15]. EOs are complex mixtures of volatile secondary metabolites, primarily terpenes and terpenoids, typically obtained by steam distillation or cold pressing of aromatic plants. Their biological effects are determined by both major and minor constituents, as well as by synergistic or antagonistic interactions between them [16].

The antimicrobial activity of essential oils has been extensively studied against a wide range of bacterial and fungal species. Many EOs have demonstrated strong inhibitory effects, particularly against Gram-positive bacteria, while Gram-negative species such as *Escherichia coli* often display greater resistance due to their outer lipopolysaccharide membrane, which acts as a permeability barrier [16,17,18]. Despite this intrinsic resistance, certain essential oils and their constituents have shown notable activity against *E. coli*, suggesting that specific components can overcome these protective mechanisms [19].

The antimicrobial effects of essential oils are primarily attributed to phenolic compounds such as thymol, carvacrol, and eugenol, which disrupt bacterial cell membranes, increase membrane permeability, and cause leakage of intracellular contents [20,21]. Other compounds, including aldehydes (e.g., cinnamaldehyde) and alcohols (e.g., linalool and terpinen-4-ol), contribute to antibacterial effects through mechanisms such as enzyme inhibition, disruption of the proton motive force, and interference with quorum sensing [22,23]. These combined or synergistic actions often enhance the overall antimicrobial efficacy of essential oils.

However, the antimicrobial potential of EOs can vary substantially depending on their chemical composition, which is influenced by factors such as plant species, chemotype, geographical origin, and extraction method [14]. This variability can result in differences in antibacterial potency even among oils derived from the same plant species. Therefore, detailed chemical characterisation and comparison of essential oils are necessary to identify the key bioactive compounds responsible for their antimicrobial activity.

Gas chromatography coupled with mass spectrometry (GC–MS) remains the most widely used analytical technique for characterising essential oils, owing to its high sensitivity and ability to detect a broad range of volatile compounds [24]. When combined with statistical methods such as principal component analysis (PCA), discriminant analysis (DA), and partial least squares (PLS), GC–MS data can be used to explore correlations between chemical composition and antimicrobial activity. These multivariate approaches enable the identification of specific marker compounds that may contribute to antibacterial properties, providing a more comprehensive understanding of the biological potential of essential oils.

Despite the increasing number of studies on essential oils, comparative analyses that integrate chemical composition with antimicrobial data across multiple oils remain limited. To address this gap, the present study aimed to evaluate the antibacterial activity of twenty essential oils and one absolute against *Escherichia coli*, determine their chemical composition using GC–MS, and identify key compounds associated with antibacterial effects through multivariate statistical analysis.

## 2. Results

### 2.1. GC–MS Analysis

All samples were analysed using GC–MS to determine their chemical composition. The results of the GC–MS analysis are presented in Table 1, Table 2 and Table 3, which list component names, retention times, and relative area percentages for constituents present at levels of at least 1%. Compounds below this threshold were excluded to focus on the main constituents likely to contribute significantly to the biological activity of the essential oils.

Table 1 shows the GC–MS results for essential oils and the absolute that exhibited good antibacterial activity, Table 2 presents those with moderate antibacterial activity, and Table 3 lists those with no antibacterial activity. The thresholds used to classify antibacterial activity were empirically defined for comparative purposes. Important compounds are marked in bold; these are compounds found only in essential oils and the absolute with good antimicrobial activity, as well as those present in at least four essential oils.

### 2.2. Antimicrobial Activity Against Escherichia coli

Antimicrobial activity against *Escherichia coli* was determined using single concentration broth inhibition test. The absorbance of the medium after incubation was measured at 600 nm, and the results are presented in Table 4. To ensure reproducibility within the scope of this study, all experiments were performed under identical conditions, including standardized inoculum preparation, inclusion of blank samples, and appropriate positive and negative controls. The activity observed for *Thymus vulgaris* essential oil was consistent with previously reported data, supporting the reliability of the assay for comparative screening purposes. Due to the uniform test concentration applied to all essential oils and the absolute, the method provides only a preliminary estimation of antibacterial potential and was therefore used as a screening approach to identify samples for further investigation. Accordingly, the results were categorised as showing good, moderate, or no antibacterial activity. Essential oils that showed similar antibacterial activity to *Thymus vulgaris* and had absorbance lower than 2.0 were ranked as good; those with absorbance higher than 2.0 but lower than the positive control were ranked as having moderate antibacterial activity; and those with absorbance higher than the positive control were ranked as having no antibacterial activity. The classification of antibacterial activity into qualitative categories was based on empirical ranges and was not supported by statistical analysis. Consequently, small differences in absorbance values should be interpreted cautiously, and the results are considered a preliminary screening method rather than an exact quantitative comparison of antibacterial potency.

### 2.3. Important Compounds

Given the limited number of samples and high feature sparsity, the analysis was focused on exploratory multivariate methods rather than predictive machine learning models. Compounds found exclusively in essential oils and the absolute with good antimicrobial activity included thymol, eugenol, and methyl atrarate. The most common compounds identified across all analysed essential oils are summarised in Table 5. Among these, linalool was the most frequently occurring constituent, detected in ten essential oils, and was particularly abundant in *Aniba roseodora* and *Dalbergia sissoo*. The antibacterial activity of certain essential oils is likely influenced by synergistic or additive interactions among multiple constituents rather than by individual compounds alone.

### 2.4. Principal Component Multivariate Analysis

Principal component analysis (PCA) was performed on the data to explore potential relationships between the chemical composition of the essential oils and their antibacterial activity. This approach was used to reduce data dimensionality and to visualise possible clustering patterns among the samples based on their compositional similarities. The first two principal components did not reveal any clear grouping according to antimicrobial activity. The compounds with the highest absolute weight values in the first two principal components were linalool, eugenol, and methyl atrarate.

Because of the high variability among samples, the principal components were subsequently used in discriminant analysis and partial least squares analysis to further examine potential patterns in the data.

### 2.5. Discriminant Analysis

Discriminant analysis was performed using both the original compositional data and the principal components derived from PCA. The analysis based on the original data did not yield meaningful results due to the high variability among samples. When applied to the principal components, some overfitting was observed; however, this was not considered critical, as the primary purpose of the analysis was to identify compounds potentially associated with antibacterial activity.

The principal components contributing most strongly to the discriminant functions were components 20, 19, 16, 9, 10, 15, 3, 11, 8, and 6, representing the five highest coefficients within each of the three discriminant functions. The numbering of the principal components reflects their order in the PCA output, where higher component numbers indicate lower explained variance but potential relevance for group discrimination. The compounds with the highest absolute coefficient values in these components: α-terpineol, methyl eugenol, cyperene, γ-muurolene, thymol, bulnesol, β-himachalene, methyl atrarate, davanone D, and linalyl acetate are listed in Table 5.

### 2.6. Partial Least Squares Multivariate Analysis

Partial least squares (PLS) analysis was applied to explore linear relationships between the chemical composition of the essential oils and their antibacterial activity. Unlike discriminant analysis, which focuses on classification, PLS identifies variables that most strongly contribute to the observed absorbance that corresponds to biological effect.

The model based on the original compositional data did not provide meaningful results due to the high variability among samples, and some overfitting was observed when using the principal components. Despite these limitations, several components showed influence patterns comparable to those obtained by discriminant analysis.

The principal components with the highest absolute loadings were components 20, 10, 12, 3, and 11, while additional contributors were identified in components 9, 8, 15, 13, and 17. The essential oil compounds most strongly associated with these components included α-terpineol, β-himachalene, citronellol, methyl atrarate, thymol, bulnesol, davanone D, cyperene, menthone, α-elemol, and methyl eugenol (Table 5).

## 3. Discussion

### 3.1. GC–MS Analysis

The chemical composition of the analysed essential oils was compared with available literature data to assess consistency and potential variability. For *Thymus vulgaris*, the composition closely matched that described in the European Pharmacopoeia [25], with thymol confirmed as the predominant compound. Other constituents were in agreement with those reported in previous studies [26,27,28,29,30,31]. Similarly, in *Aniba rosaeodora*, linalool was consistently identified as the main component across various sources [32,33,34,35,36,37,38,39,40], and the overall composition showed good agreement with published data. The composition of *Syzygium aromaticum* also corresponded well with the literature, which consistently identifies eugenol as the dominant compound, along with caryophyllene and eugenyl acetate [41,42,43,44].

In the case of *Evernia prunastri*, where an absolute was analysed, methyl atrarate (atraric acid) was confirmed as a key constituent, consistent with previous reports [45]. *Pimenta dioica* showed a composition consistent with published data [46,47,48,49,50,51,52], as did *Bulnesia sarmientoi* [53,54,55]. For *Pelargonium graveolens*, most compounds matched literature sources [56,57,58,59,60], although phenethyl alcohol, nerol, lavandulyl acetate, and citronellyl acetate had not been previously reported as major components.

Among the remaining essential oils, including *Agathosma betulina* [61,62,63], *Thymus hiemalis* [64], *Canarium luzonicum* [65], *Illicium verum* [66], *Cymbopogon martini* [67], *Citrus bergamia* [68,69,70,71,72], *Salvia sclarea* [73,74,75], and *Cananga odorata* [76,77,78], the main constituents were also in good agreement with literature data, although some variability in major compounds was observed for *C. odorata* [79,80].

For *Ravensara aromatica*, the findings of this study differed somewhat from those of [81], which reported eucalyptol as the dominant compound. In the analysed sample, eucalyptol was present in lower amounts, while limonene and sabinene were more prominent. However, other studies [82,83] have described chemotypes rich in limonene and methyl eugenol, suggesting natural chemical variability.

The composition of *Matricaria recutita* analysed in this study was generally consistent with the literature [84,85], in which farnesene and bisabolol oxides predominate. Isobutyl angelate was also detected, which may reflect natural intraspecific variation.

For *Cedrus atlantica*, *Artemisia pallens*, and *Cyperus scariosus*, most of the detected compounds matched those reported in the literature [86,87,88]. In contrast, no literature data on the essential oil composition could be found for *Dalbergia sissoo*, highlighting a gap in current phytochemical knowledge for this species.

### 3.2. Antimicrobial Activity Against Escherichia coli

Gram-negative bacteria are generally more resistant to essential oils (EOs) than Gram-positive species such as *Staphylococcus aureus*, mainly due to the presence of an outer lipopolysaccharide membrane that limits the penetration of hydrophobic molecules [16,89,90,91,92]. Nevertheless, several EOs rich in phenolic constituents, particularly *Thymus vulgaris* and *Syzygium aromaticum*, have demonstrated significant antibacterial activity against *Escherichia coli* [93,94], with eugenol also known to inhibit *E. coli* biofilm formation [94,95].

**Figure 1:**
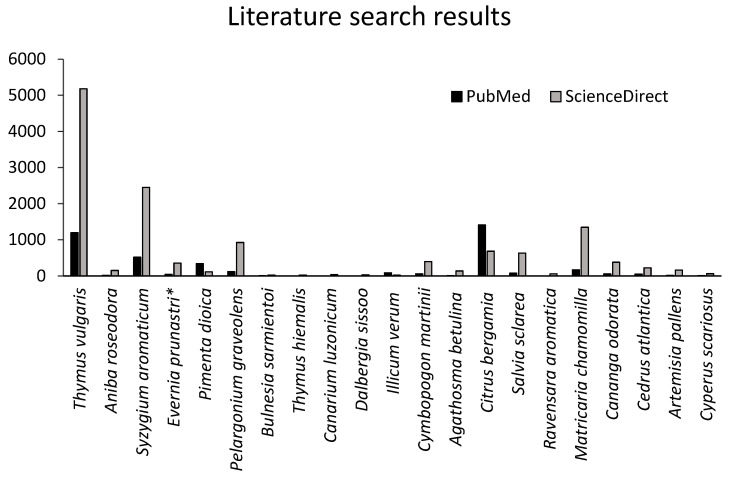
Number of results found in the research databases PubMed and ScienceDirect using the name of the plant with the term “essential oil” or “extract” (*) on 1 October 2025.

In the present study, five samples: EOs of *T. vulgaris*, *A. rosaeodora*, *S. aromaticum*, *P. dioica*, and the absolute of *E. prunastri* showed the strongest antibacterial activity. The EOs of *C. atlantica*, *A. pallens*, and *C. scariosus* exhibited no activity, while the remaining oils demonstrated moderate inhibition. Figure 1 illustrates the relative frequency of publications for each plant species tested, showing that *E. prunastri* and several other oils investigated in this work have been poorly explored compared with *T. vulgaris* and *S. aromaticum*.

The results are partially consistent with previous studies. In [96], *T. vulgaris* showed antibacterial activity comparable to ampicillin, while *A. rosaeodora* exhibited similar inhibitory effects in both studies. *Cymbopogon martinii* also showed strong activity in [96], but only moderate inhibition was observed in our experiments. *C. bergamia* was inactive in their study but showed moderate activity in ours, whereas *C. atlantica* exhibited no inhibition in either. Direct comparison is limited by the absence of EO compositional data in [96], which is critical given the natural variability of essential oils. In [97], which tested 52 EOs (nine overlapping with our selection), *T. vulgaris* exhibited the highest antibacterial activity, and both *A. rosaeodora* and *S. aromaticum* demonstrated strong effects against *E. coli* in both studies. *C. martinii* showed strong activity in [97] but only moderate in our work, while *P. graveolens*, *C. bergamia*, and *C. odorata* showed moderate activity in both. *S. sclarea* displayed poor activity in [97] but moderate inhibition in our results, and *C. atlantica* consistently showed no effect. As in [96], the lack of compositional analysis in [97] limits direct interpretation.

Similar trends were reported for individual EOs in other studies. *P. dioica* exhibited antibacterial activity against *E. coli*, with eugenol identified as the main active compound [48]. *T. hyemalis* demonstrated antibacterial effects in two studies [64,98]; notably, the chemotype with the highest proportion of eucalyptol (5.4%) was the least active, whereas our EO contained 30.6% eucalyptol. *C. luzonicum*, rich in limonene and α-phellandrene, showed moderate activity against *E. coli* in [99], aligning with our findings. *I. verum*, with a composition similar to ours, displayed activity against Gram-negative bacteria [100], including E. coli [101]. In [95], *A. betulina* exhibited a 6 mm inhibition zone against *E. coli*, markedly smaller than that of *T. vulgaris* (39.1 mm), which corresponds to our results. *S. sclarea* showed some antibacterial activity in [75], and microscopy in another study revealed membrane damage caused by the EO [102]. *R. aromatica* had an MIC of 4% compared to 0.5% for thyme, with a different composition dominated by eucalyptol (19.68%) [103]. *M. recutita*, containing high levels of *trans*-β-farnesene and α-bisabolol oxide A, exhibited antimicrobial activity against *E. coli* in two studies [84,104], consistent with our observations. *C. odorata* displayed good antimicrobial activity in [76], while *A. pallens* showed no antibacterial activity in our work but was reported as active in some studies where oils were rich in davanone and bicyclogermacrene [87,105,106].

Table 5 summarises the key compounds identified by comparing the chemical composition of the essential oils and absolutes with their antibacterial activity. Compounds found to have strong antibacterial effects were thymol, eugenol, and methyl atrarate, in good agreement with previous reports. These were present in high concentrations exclusively in oils and the absolute with strong antibacterial activity (“only in good”). Compounds detected in several EOs with moderate activity included linalool, limonene, β-caryophyllene, eucalyptol, α-pinene, β-pinene, *p*-cymene, terpinene-4-ol, myrcene, geraniol, geranyl acetate, α-terpineol, γ-muurolene, γ-terpinene, α-humulene, and sabinene, occurring in at least three EOs. These were absent in oils with no activity, except for β-pinene (1.1% in *C. scariosus* EO). Although these constituents have not been proven to possess strong antibacterial effects individually, they may play important synergistic roles in enhancing overall activity.

Several reviews have discussed these interactions in detail. In [107], eugenol was identified as a key marker of antibacterial activity, while linalyl acetate, α-pinene, and β-pinene were associated with weaker effects. EOs from thyme, bergamot, clary sage, clove, elemi, and pimento exhibited good minimum inhibitory concentrations (MICs) against various bacteria, whereas those from star anise showed poor MIC values, although *E. coli* was not included in that study. In [16], antibacterial properties were mainly attributed to major constituents such as linalool, *trans*-cinnamaldehyde, carvacrol, thymol, γ-terpinene, *p*-cymene, α-pinene, β-pinene, bornyl acetate, camphor, eucalyptol, α-thujone, eugenol, and eugenyl acetate. Some of these compounds were also identified in our study; however, not all exhibited strong effects. Components such as α-terpineol, terpinen-4-ol, linalool, linalyl acetate, menthol, α-pinene, and limonene [94], found in *S. aromaticum*, *P. dioica*, and *T. vulgaris*, have been reported to be effective against *E. coli* biofilms [94].

Several of the essential oils analysed in this work—particularly *E. prunastri* and *C. scariosus*—have rarely been investigated for antibacterial activity in the published literature. Figure 1 illustrates this knowledge gap. Overall, a lack of reproducibility among studies on EO antibacterial activity is evident, likely due to the lipophilic and volatile nature of EOs and natural variation in their chemical composition. Standardised methods for analysis and microbiological evaluation, as well as publication of negative results, are essential to improve reproducibility and data transparency in EO research.

### 3.3. Multivariate Identification of Compounds Associated with Antibacterial Activity

Multivariate statistical analyses (PCA, DA, and PLS) were applied to explore relationships between the chemical composition of essential oils and their antibacterial activity. PCA, PLS, and DA are well-established multivariate approaches widely used for data exploration, regression, and classification. These approaches enabled the identification of compounds most strongly associated with the observed biological effects. The use of more complex machine learning classifiers was not pursued due to the small sample size and high chemical heterogeneity, which would likely result in overfitting and limited interpretability.

Compounds identified as important by multivariate analysis approaches—PCA, DA, and PLS—are listed in Table 5, marked as “yes” in the corresponding column. Thymol and methyl atrarate were recognised as key compounds, while among the compounds highlighted by the first approach, only α-terpineol and γ-muurolene were confirmed as important. Additional compounds of interest included those present as the main constituents in essential oils with no antibacterial activity: cyperene in *Cyperus scariosus*, β-himachalane in *Cedrus atlantica*, and davanone D in *Artemisia pallens*. These compounds were associated with the absence of antibacterial activity and could therefore be of interest for future studies exploring their potential influence on bacterial survival in the presence of other essential oil constituents. Other important compounds identified through multivariate analysis were also major constituents in certain essential oils: bulnesol in *Bulnesia sarmientoi*, linalyl acetate in *Salvia sclarea*, citronellol in *Pelargonium graveolens*, and menthone in *Agathosma betulina*. Compounds such as methyl eugenol, linalyl acetate, and α-elemol were identified because they commonly co-occur with limonene and can therefore help distinguish samples where limonene is more generally present. Since the antibacterial activity of limonene-rich essential oils varies considerably, these co-occurring compounds may play an important role and their potential contribution to antibacterial activity should be further investigated.

Overall, the multivariate analysis identified compounds that are more important for differentiating among essential oils, rather than for their absolute antibacterial activity. The most commonly present compounds were not detected as important, as they do not contribute to differentiation. This is illustrated by limonene, one of the most widespread compounds in the analysed essential oils, which was not identified as significant, whereas several accompanying constituents were emphasised instead.

Multivariate analysis provided some insight into the highly diverse data obtained in this study. However, due to this diversity, the results raise more research questions than they answer. In general, multivariate approaches yield clearer patterns when applied to datasets containing numerous comparable samples, which was not the case here. Under such conditions, the true potential of multivariate analysis can be harnessed to reveal subtle differences among similar samples and to identify meaningful attributes for group differentiation.

In the present experimental setup, several compounds were identified that are specific to individual essential oils or act as accompanying constituents. These findings merit further investigation, particularly regarding potential synergistic effects and the possible role of these compounds in supporting bacterial survival under stress conditions.

A particularly interesting direction for further exploration of synergism would be a detailed comparison of *Aniba roseodora* and *Dalbergia sissoo* essential oils, which share similar compositions but very different antibacterial activities.

## 4. Materials and Methods

### 4.1. Samples

Samples tested are given in Table 6. The table provides an overview of the essential oils and the absolute included in this study, together with their plant sources and suppliers. These samples were selected to represent a diverse range of botanical families and chemical compositions for comparative evaluation of their antibacterial properties.

### 4.2. GC–MS Analysis

Samples of essential oils and absolute for GC-MS analyses were prepared as 1% solutions in n-hexane for gas chromatography–mass spectrometry (SupraSolv, Merck, Darmstadt, Germany). GC–MS analyses were performed using a GCMS-QP2010 Ultra system (Shimadzu Corporation, Kyoto, Japan) and the data libraries NIST14 (National Institute of Standards and Technology, Gaithersburg, MD, USA) and FFNSC3 (Shimadzu Corporation, Kyoto, Japan). The GC system was equipped with an Rxi-5SilMS capillary column (30 m length, 0.25 mm internal diameter, 0.25 µm film thickness; Restek, Bellefonte, PA, USA). The carrier gas was helium with a constant column flow rate of 1 mL/min. The mass spectrometer ionisation energy was 70 eV, the ion source temperature was 200 °C, and the detector voltage was 1 kV. The injection volume was 1 μL, with a split ratio of 1:100. The injection port was set to 250 °C, and the interface temperature to 300 °C; a 3.5 min solvent delay was used. A full scan was recorded in the mass range 40–400 *m*/*z* with a scanning frequency of 5 Hz. The temperature program began at 40 °C, increased to 220 °C at 3 °C/min, and was held at 220 °C for 15 min (total analysis time 75 min).

Samples were analysed in triplicate and averaged. Peaks that did not appear in all three replicates were excluded from the list of compounds. Mass spectra were initially compared with the NIST and FFNSC libraries. Identifications were then manually verified through visual inspection of the spectra. In cases where multiple matches were obtained, the highest-scoring library hits were considered, with priority given to FFNSC matches. Retention indices were also compared with library values to support the identifications. Linalool was confirmed by comparison with an authentic standard, due to its frequent misidentification with linalyl anthranilate. In cases where multiple high-confidence matches were obtained and a clear assignment could not be made, literature data were consulted to confirm the correct compound. The list of detected compounds above 1% and their average area percentage were used for further analysis of the results (Table S1).

### 4.3. Antimicrobial Activity Against Escherichia coli

Samples of twenty essential oils and one absolute were dissolved in DMSO (Sigma-Aldrich Co., St. Louis, MO, USA) to obtain a final essential oil or absolute concentration of 10% (*v*/*v*). Each solution was then filtered through Millex-GV syringe filters (0.22 μm pore size). This filtration step was performed to remove any fungi (spores and mycelia) and bacteria potentially present in the essential oils in DMSO solutions. The prepared solutions were stored at 4 °C until testing.

Tetracycline (Sigma-Aldrich Co., St. Louis, MO, USA) was weighed and transferred into an Eppendorf tube and dissolved in a solvent mixture with isopropyl alcohol (Sigma-Aldrich Co., St. Louis, MO, USA) (water:isopropyl alcohol = 20:1, *v*/*v*) to prepare a concentrated standard solution (20 mg/mL).

Antimicrobial activity against *Escherichia coli* ATCC 25922 was determined using the single concentration broth inhibition test. The growth method of *E. coli* in liquid culture served as the primary method for evaluating the antimicrobial activity of the essential oils. Medium was prepared from LB Broth (Sigma-Aldrich Co., St. Louis, MO, USA) dissolved in water and autoclaved. 5 mL of sterile liquid LB medium was pipetted into sterile test tubes, followed by the addition of 0.5 mL of essential oil or absolute samples dissolved in DMSO, and 0.1 mL of an overnight suspension of *E. coli*.

The positive control consisted of LB medium with *E. coli* (0.1 mL overnight suspension) and 0.5 mL of sterile, pre-filtered DMSO. The negative control consisted of LB medium, 0.1 mL of the overnight *E. coli* culture, and 0.05 mL of the tetracycline solution (see Preparation of tetracycline standard solution).

Samples were incubated in a shaker incubator at 800 rpm for 24 h at 37 °C.

Bacterial growth inhibition was measured spectrophotometrically at 600 nm. The absorbance values were corrected by subtracting the baseline absorbance of the negative control. The positive control served as a reference for normal bacterial growth without any antimicrobial agents (either essential oil, absolute or antibiotic).

The experiment was conducted in duplicate, and the final absorbance values were calculated as the average of the two readings, after subtracting the absorbance of the negative control.

### 4.4. Multivariate Analysis

All multivariate statistical analyses were performed in GNU Octave (version 10.2.0), an open-source numerical computing environment. The input data for the multivariate analyses consisted of a data matrix of 21 samples of essential oils and absolute, and the relative concentrations (above 1%) of 117 compounds identified by GC–MS analysis. For predicted variables, results of antibacterial activity were used, absorbance values for partial least squares analysis and three groups for good, moderate and no antibacterial activity for discriminant analysis.

Principal component analysis was performed using the “pca” function, and 20 principal components were retained. Feature relevance in PCA was assessed based on the absolute values of the loading coefficients, which describe the contribution of each compound to the principal components.

Discriminant analysis was performed using the “fitcdiscr” function on both the original dataset (117 variables) and on the reduced dataset consisting of the first 20 principal components. Feature relevance was evaluated using the absolute values of the discriminant coefficients, which reflect the contribution of each variable or principal component to class separation. Three discriminant functions were obtained corresponding to the three activity classes.

Partial least squares analysis was performed using the “plsregress” function on both the original dataset (117 variables) and the reduced dataset (20 principal components). Feature relevance was assessed based on the absolute values of the regression coefficients, which indicate the strength of the relationship between each variable (or principal component) and the antibacterial response.

Across all methods, variables with higher absolute coefficient values were considered to have a stronger influence on the multivariate models and were therefore interpreted as more relevant for antibacterial activity.

The purpose of these analyses was variable relevance assessment and chemical interpretation rather than the development of optimized predictive models.

## 5. Conclusions

This study evaluated the antimicrobial activity of 20 essential oils and one absolute against *Escherichia coli*. The results revealed substantial variability in antibacterial efficacy, reflecting the chemical complexity of essential oils and the diverse mechanisms through which their constituents exert biological effects.

Essential oils of *Thymus vulgaris*, *Aniba rosaeodora*, *Syzygium aromaticum*, *Pimenta dioica* and absolute of *Evernia prunastri* exhibited strong antimicrobial activity, suggesting that they contain potent bioactive compounds capable of inhibiting the growth of *E. coli*. In contrast, essential oils of *Cedrus atlantica*, *Artemisia pallens*, and *Cyperus scariosus* showed no activity, which could be due to the absence of effective antimicrobial compounds or the presence of these compounds in insufficient concentrations. The remaining oils demonstrated moderate activity, which may be attributed to partial effectiveness of their constituents or synergistic interactions between multiple compounds.

Chemical analysis revealed that three compounds, thymol, eugenol, and methyl atrarate, were present exclusively in the most active samples, indicating their potential importance in antimicrobial action. Sixteen additional compounds (linalool, limonene, β-caryophyllene, eucalyptol, α-pinene, β-pinene, *p*-cymene, terpinen-4-ol, myrcene, geraniol, geranyl acetate, α-terpineol, γ-muurolene, γ-terpinene, α-humulene, and sabinene) were associated with moderate activity, possibly contributing to partial inhibition or enhancing the effects of primary active constituents. Conversely, several compounds identified mainly in inactive oils appeared to lack significant antibacterial properties, suggesting that not all constituents contribute to biological activity.

Multivariate analysis provided additional insight into the dataset, identifying compounds of potential relevance to antibacterial activity. However, these findings should be interpreted with caution due to the high variability among samples and the limited number of comparable data points.

While the study provides useful insights into the antimicrobial potential of essential oils against *E. coli*, it is limited by its focus on a single bacterial species. Testing the same oils against a broader range of microorganisms, including Gram-positive bacteria and fungi, would provide a more comprehensive understanding of their antimicrobial spectrum. Furthermore, quantitative measurements such as minimum inhibitory concentrations (MICs) and assessments of cytotoxicity would be valuable in evaluating their potential for therapeutic or preservative use.

In conclusion, the results suggest that certain essential oils and specific compounds within them hold promise as natural antimicrobial agents. However, further studies are necessary to better understand their mechanisms of action, the role of compound interactions, and their efficacy in different biological contexts.

## Figures and Tables

**Table 1:** GC-MS results of essential oils and absolute (marked with *), which showed good antibacterial activity. Compounds above 1% are listed with retention times in minutes and relative area percentages. Important compounds are marked in bold.

*Thymus vulgaris*			*Aniba roseodora*			*Syzygium aromaticum*			Evernia prunastri *			*Pimenta dioica*		
RetT	Compound	%	RetT	Compound	%	RetT	Compound	%	RetT	Compound	%	RetT	Compound	%
25.9	**thymol**	50.0	17.0	**linalool**	86.2	28.6	**eugenol**	85.1	42.5	**methyl atrarate**	100.0	28.5	**eugenol**	71.6
13.3	***p*** **-cymene**	17.4	13.7	**eucalyptol**	4.3	35.3	eugenyl acetate	7.3				30.6	methyl eugenol	12.5
14.9	γ-terpinene	7.2	24.0	**geraniol**	2.1	31.4	***trans*** **-caryophyllene**	5.4				31.4	***trans*** **-caryophyllene**	8.0
16.9	**linalool**	4.2	9.3	**α** **-pinene**	1.9							32.8	α-humulene	1.7
26.2	carvacrol	3.3	13.5	**limonene**	1.9									
20.7	**terpinen-4-ol**	2.6	11.2	**β-pinene**	1.7									
20.2	borneol	1.9												
31.4	***trans*** **-caryophyllene**	1.8												
19.0	camphor	1.4												
13.0	α-terpinene	1.2												
11.8	**myrcene**	1.2												
13.7	**eucalyptol**	1.2												
10.0	camphene	1.1												

**Table 2:** GC-MS results of essential oils exhibiting moderate antibacterial activity. Compounds present at concentrations above 1% are listed with their retention times in minutes and relative area percentages. Important compounds are marked in bold. NI are not identified compounds, most important *m*/*z* signals are added.

***Pelargonium graveolens***			***Bulnesia sarmientoi***				***Thymus hiemalis***			***Canarium luzonicum***				***Dalbergia sissoo***		
**RetT**	**Compound**	**%**	**RetT**	**Compound**		**%**	**RetT**	**Compound**	**%**	**RetT**	**Compound**		**%**	**RetT**	**Compound**	**%**
23.0	citronellol	35.7	41.2	bulnesol		40.3	13.6	**eucalyptol**	30.6	13.5	**limonene**		50.1	16.9	**linalool**	79.8
24.0	geraniol	14.8	38.6	guaiol		34.9	19.0	camphor	9.3	36.6	α-elemol		25.7	21.3	**α-terpineol**	6.1
17.4	phenethyl alcohol	10.4	40.7	β-eudesmol		7.5	16.9	**linalool**	8.6	12.4	α-phellandrene		6.7	13.6	**eucalyptol**	3.8
29.7	**geranyl acetate**	7.1	39.9	δ-eudesmol		3.2	10.0	camphene	5.9	11.0	sabinene		4.7	16.3	NI: 71, 43, 68, 41	3.6
25.1	citronellyl formate	4.6	39.5	eudesmol (10-epi-δ)		1.8	13.3	***p*-cymene**	4.8	13.3	***p*-cymene**		3.2	23.9	geraniol	2.7
28.9	neryl acetate	3.6	39.0	guaiol		1.6	20.2	borneol	4.6	21.3	**α-terpineol**		2.4	13.5	**limonene**	1.7
22.8	nerol	2.9	39.1	rosifoliol		1.2	9.3	**α-pinene**	3.7							
16.9	**linalool**	2.8					14.4	*trans*-β-ocimene	3.1							
19.9	isomenthone	2.8					11.8	myrcene	3.0							
39.5	δ-eudesmol	2.7					21.3	**α-terpineol**	2.6							
26.2	lavandulyl acetate	1.4					11.2	**β-pinene**	2.4							
28.5	citronellyl acetate	1.1					31.4	***trans*-caryophyllene**	2.1							
							24.7	geranial	2.0							
							14.9	δ-terpinene	1.9							
							13.5	**limonene**	1.8							
							20.6	terpinen-4-ol	1.8							
							11.0	sabinene	1.4							
							23.4	neral	1.4							
							34.5	bicyclogemacrene	1.1							
***Illicum verum***			***Cymbopogon martinii***				***Agathosma betulina***			***Citrus bergamia***				***Salvia sclarea***		
**RetT**	**Compound**	**%**	**RetT**	**Compound**		**%**	**RetT**	**Compound**	**%**	**RetT**	**Compound**		**%**	**RetT**	**Compound**	**%**
25.6	*trans*-anethole	90.92	24.1	geraniol		76.87	19.5	menthone	32.5	13.5	**limonene**		37.0	24.0	linalyl acetate	62.2
21.5	estragole	3.10	29.7	**geranyl acetate**		9.63	13.5	**limonene**	20.0	24.0	linalyl acetate		31.1	16.9	**linalool**	23.3
13.5	**limonene**	1.22	31.3	***trans*-caryophyllene**		3.54	19.9	isomenthone	11.5	16.9	**linalool**		11.3	21.3	**α-terpineol**	4.5
			16.9	**linalool**		2.80	26.0	diosphenol	8.5	11.2	**β-pinene**		6.7	29.7	**geranyl acetate**	2.4
			13.5	**limonene**		1.22	24.5	pseudodiosphenol	7.4	14.9	γ-terpinene		6.6	31.3	***trans*-caryophyllene**	1.5
							23.3	pulegone	5.3	9.3	**α-pinene**		1.1	28.8	neryl acetate	1.2
							13.7	**eucalyptol**	3.8					33.9	**γ-muurolene**	1.2
							9.3	**α-pinene**	2.5							
							11.8	**myrcene**	1.4							
							24.0	NI: 43, 69, 112, 70, 55	1.2							
							20.7	**terpinen-4-ol**	1.1							
***Ravensara aromatica***					***Matricaria chamomilla***							***Cananga odorata***				
**RetT**	**Compound**			**%**	**RetT**	**Compound**					**RetT**	**RetT**	**Compound**			**RetT**
13.5	**limonene**			16.0	14.6	isobutyl angelate					20.9	33.9	**γ-muurolene**			18.9
11.0	sabinene			9.8	32.9	*trans*-β-farnesene					15.7	19.8	benzyl acetate			11.9
30.6	methyl eugenol			8.2	44.1	α-bisabolol oxide a					15.5	35.0	*trans*, *trans*-α-farnesene			8.8
31.3	***trans*-caryophyllene**			6.1	19.3	2-methylbutyl angelate					11.5	16.9	**linalool**			8.1
13.3	***p*-cymene**			5.3	15.2	(2z)-hexenyl tiglate					5.3	44.7	benzyl benzoate			7.2
16.9	**linalool**			5.0	40.7	α-bisabolol oxide b					3.5	13.1	*p*-methyl anisole			6.6
33.9	γ-muurolene			4.8	41.7	α-bisabolone oxide a					3.4	31.4	***trans*-caryophyllene**			5.0
9.3	**α-pinene**			4.4	18.7	trans-pinocarveol					3.3	29.7	**geranyl acetate**			4.5
20.6	**terpinen-4-ol**			3.9	19.1	isoamyl angelate					2.9	16.5	clorius			4.2
36.6	elemicin			3.3	8.6	isobutyl isobutyrate					2.0	32.4	*trans*-cinnamyl acetate			4.0
21.4	estragole			3.3	13.0	isopentyl isobutyrate					1.3	47.2	farnesyl acetate			2.8
12.5	δ-3-carene			3.1	33.9	**γ-muurolene**					1.2	48.3	benzyl salicylate			2.3
11.1	**β-pinene**			2.7								32.9	α-humulene			1.9
12.9	α-terpinene			2.3								35.5	δ-cadinene			1.8
11.7	**myrcene**			1.8								40.7	cadin-4-en-10-ol			1.5
13.6	**eucalyptol**			1.5								8.9	prenyl acetate			1.1
10.0	camphene			1.4												
32.8	α-humulene			1.3												
29.5	α-copaene			1.2												

**Table 3:** GC-MS results of essential oils that did not show antibacterial activity. Compounds above 1% are listed with retention times in minutes and relative area percentage. Important compounds are marked in bold. NI are not identified compounds, most important *m*/*z* signals are added.

*Cedrus atlantica*			*Artemisia pallens*			*Cyperus scariosus*		
RetT	Compound	%	RetT	Compound	%	RetT	Compound	%
34.8	β-himachalene	47.2	37.9	davanone D	56.4	30.7	cyperene	26.3
32.6	α-himachalene	17.2	34.6	bicyclogermacrene	8.5	33.2	rotundene	7.6
33.8	γ-himachalene	10.5	33.4	*trans*-ethyl-cinnamate	4.1	42.3	cyperotundone	5.6
45.1	trans-α-atlantone	2.6	34.9	NI: 109, 43, 124	3.1	29.6	α-copaene	4.8
34.0	himachalene-1,4-diene	2.1	34.3	β-selinene	1.6	34.5	eremophilene	3.8
35.5	δ-cadinene	1.8	35.7	NI: 109, 43, 124	1.6	38.0	caryophyllene oxide	3.0
35.7	γ-dehydro-ar-himachalene	1.6	40.3	epi-α-cadinol	1.6	34.3	β-selinene	2.7
39.1	β-himachalene oxide	1.4	37.8	spathulenol	1.6	41.5	mustakone	2.5
36.4	*trans*-α-bisabolene	1.2	37.1	davanone B	1.5	44.0	NI: 93, 91, 218, 147, 121	2.3
			29.5	*cis*-ethyl-cinnamate	1.2	35.5	α-maaliene	2.2
						39.5	NI: 175, 218, 147	2.2
						33.6	γ-gurjunene	1.9
						36.1	isospathulenol	1.5
						34.6	α-selinene	1.4
						34.9	α-bulnesene	1.4
						34.1	aristochelene	1.4
						29.0	valerenyl acetate	1.3
						40.8	NI: 93, 91, 107, 41	1.3
						11.2	**β-pinene**	1.1
						42.4	NI: 123, 81, 95, 124, 107	1.1
						32.6	α-guaiene	1.0

**Table 4:** Results of absorbance measurements for 20 essential oils and absolute (marked with *). Negative and positive controls are indicated in bold.

Sample Tested	Absorbance
**Negative control (tetracycline)**	0.00
*Thymus vulgaris*	0.67
*Aniba roseodora*	0.89
*Syzygium aromaticum*	1.42
Evernia prunastri *	1.47
*Pimenta dioica*	1.91
*Pelargonium graveolens*	3.09
*Bulnesia sarmientoi*	3.16
*Thymus hiemalis*	3.83
*Canarium luzonicum*	3.87
*Dalbergia sissoo*	3.90
*Illicum verum*	3.92
*Cymbopogon martinii*	4.00
*Agathosma betulina*	4.13
*Citrus bergamia*	4.18
*Salvia sclarea*	4.30
*Ravensara aromatica*	4.47
*Matricaria chamomilla*	4.55
*Cananga odorata*	4.58
**Positive control (DMSO)**	4.66
*Cedrus atlantica*	4.82
*Artemisia pallens*	4.88
*Cyperus scariosus*	4.91

**Table 5:** Compounds identified as important in three different analyses. The first column lists compounds present only in oils with good antimicrobial activity and those common to all samples studied; the second column lists those identified by discriminant analysis; the third column lists those identified by PLS.

Compound Name	Number of Occurrences (GC-MS)	Shown to Be Important in DA	Showed to Be Important in PLS
thymol	Only in good	yes	yes
eugenol	Only in good	-	-
methyl atrarate	Only in good	yes	yes
linalool	10	-	-
limonene	9	-	-
*trans*-caryophyllene	8	-	-
eucalyptol	6	-	-
α-pinene	5	-	-
β-pinene	5	-	-
*p*-cymene	4	-	-
terpinen-4-ol	4	-	-
myrcene	4	-	-
geraniol	4	-	-
geranyl acetate	4	-	-
α-terpineol	4	yes	yes
γ-muurolene	4	yes	-
γ-terpinene	3	-	-
α-humulene	3	-	-
sabinene	3	-	-
methyl eugenol	<3	yes	yes
cyperene	<3	yes	yes
bulnesol	<3	yes	yes
β-himachalene	<3	yes	yes
davanone D	<3	yes	yes
linalyl acetate	<3	yes	-
citronellol	<3	-	yes
menthone	<3	-	yes
α-elemol	<3	-	yes

**Table 6:** Samples of essential oils and absolute (marked with *) tested for antibacterial activity against *E. coli*.

Latin Name	Common Name	Manufacturer, Lot (County of Origin)	Plant Part
*Thymus vulgaris*	common thyme	Dagmar Köhler, Weseler Strasse 2, Alpen, Germany. Lot 7371 (France)	-
*Aniba rosaeodora*	rosewood	Aliacura, Cuxhavener Strasse 263, Hamburg, Germany. Lot 150000213 (Brasilien)	wood
*Syzygium aromaticum*	clove tree	Dagmar Köhler, BaccaraRose, Weseler Strasse 2, Alpen, Germany. Lot 29970 (Indonesien)	flowers
Evernia prunastri *	oakmoss	absolute 15% in ethanol, Dagmar Köhler, Weseler Strasse 2, Alpen, Germany. Lot 5944 (Morocco)	moss
*Pimenta dioica*	allspice tree	Dagmar Köhler, Weseler Strasse 2, Alpen, Germany. Lot 11518 (Jamaica)	berries
*Pelargonium graveolens*	rose geranium	Caelo, Caesar&Loretz, GmbH Herderstrasse 31, Hilden, Germany. Lot 12354108	-
*Bulnesia sarmientoi*	guaiacwood	Dagmar Köhler, Weseler Strasse 2, Alpen, Germany. Lot 8542 (Paraguay)	wood
*Thymus hiemalis*	winter thyme	Dagmar Köhler, BaccaraRose, Weseler Strasse 2, Alpen, Germany. Lot 33636 (Spain)	-
*Canarium luzonicum*	canarium elemi	Dragonspice Naturwaren, Im Staudfuß 4, Reutlingen, Germany. Lot W 20-08 (France)	resin
*Dalbergia sissoo*	indian rosewood	Manske, Geschwister-Scholl-Straße 7, Schwäbisch Hall, Germany. Lot 2014 102287 (India)	wood
*Illicium verum*	star anise	Lex, Vanganelska cesta 26, Koper, Slovenia. Lot 103190	fruits
*Cymbopogon martinii*	palmarosa	Dagmar Köhler, Weseler Strasse 2, Alpen, Germany. Lot 4538 (Nepal)	-
*Agathosma betulina*	buchu	Dagmar Köhler, Weseler Strasse 2, Alpen, Germany. Lot 7123 (South Africa)	leaves
*Citrus bergamia*	bergamot orange	Dagmar Köhler, BaccaraRose, Weseler Strasse 2, Alpen, Germany. Lot 26562 (Italy)	peel
*Salvia sclarea*	clary sage	Lex, Vanganelska cesta 26, Koper, Slovenia. Lot 10200052	flowering stems
*Ravensara aromatica*	ravensara	Dagmar Köhler, Weseler Strasse 2, Alpen, Germany. Lot 19698 (Madagascar)	leaves
*Matricaria chamomilla*	wild chamomiles	In-house production, University of Ljubljana, Faculty of Pharmacy, Department of Pharmaceutical Biology, Aškerčeva cesta 7, Ljubljana, Slovenia. (Egipt)	flowers
*Cananga odorata*	ylang ylang	Caelo, Caesar&Loretz, GmbH Herderstrasse 31, Hilden, Germany. Lot 12040607	-
*Cedrus atlantica*	atlas cedar	Dagmar Köhler, Weseler Strasse 2, Alpen, Germany. Lot 6066 (Morocco)	wood
*Artemisia pallens*	davana	Behawe, Zum Sporkfeld 48, Rietberg, Germany. Lot 111508 (India)	leaves and flowers
*Cyperus scariosus*	cypriol	Dagmar Köhler, Weseler Strasse 2, Alpen, Germany. Lot K00548 (India)	roots

## Data Availability

The data presented in this study are available from the corresponding author upon reasonable request.

## Supplementary Materials

The following supporting information can be downloaded at: https://www.mdpi.com/article/10.3390/molecules31020207/s1, Table S1: Chemical composition of essential oils and absolutes (marked with *) analyzed by GC–MS.

## Abbreviations

The following abbreviations are used in this manuscript:

GC-MS	Gas chromatography coupled with mass spectrometry
EO	Essential oil
MIC	Minimum inhibitory concentration
PCA	Principal components analysis
DA	Discriminant analysis
PLS	Partial least squares analysis
