# Supplementary material for: Multivariate Characterization of Essential Oils for Their Antibacterial Activity Against Escherichia coli: A Data-Driven Interpretation of Experimental Results
Source: Molecules. 2026 Jan 7;31(2):207. doi: 10.3390/molecules31020207 (PMC12844431; doi:10.3390/molecules31020207)
Supplement: Supplementary file 1 [file molecules-31-00207-s001.zip › molecules-4013980-supplementary.docx]

**Supplementary Material Table S1.** Chemical composition of essential oils and absolutes (marked with *) analyzed by GC–MS. Compounds present at concentrations ≥ 1% are listed. Retention times (RetT, min) and relative area percentages (%) are reported. Compound identification was performed by comparison of mass spectra and retention indices (RI) with reference data (RI (Db), database values; RI (Ms), experimentally determined values). For unidentified compounds, the most intense ions in the mass spectrum are listed, along with their relative intensities in parentheses.

| ***Thymus vulgaris*** | | | | |
| --- | --- | --- | --- | --- |
| *RetT* | *RI (Ms)* | *RI (Db)* | *Compound* | *%* |
| 25.9 | 1292 | 1293 | **thymol** | 50.0 |
| 13.3 | 1024 | 1025 | ***p*-cymene** | 17.4 |
| 14.9 | 1057 | 1058 | γ-terpinene | 7.2 |
| 16.9 | 1100 | 1104 | **linalool** | 4.2 |
| 26.2 | 1299 | 1300 | carvacrol | 3.3 |
| 20.7 | 1179 | 1184 | **terpinen-4-ol** | 2.6 |
| 20.2 | 1170 | 1173 | borneol | 1.9 |
| 31.4 | 1417 | 1424 | ***trans*-caryophyllene** | 1.8 |
| 19.0 | 1145 | 1149 | camphor | 1.4 |
| 13.0 | 1016 | 1018 | α-terpinene | 1.2 |
| 11.8 | 989 | 991 | **myrcene** | 1.2 |
| 13.7 | 1031 | 1032 | **eucalyptol** | 1.2 |
| 10.0 | 947 | 953 | camphene | 1.1 |
| ***Aniba roseodora*** | | | | |
| *RetT* | *RI (Ms)* | *RI (Db)* | *Compound* | *%* |
| 17.0 | 1100 | 1104 | **linalool** | 86.2 |
| 13.7 | 1029 | 1032 | **eucalyptol** | 4.3 |
| 24.0 | 1249 | 1244 | **geraniol** | 2.1 |
| 9.3 | 930 | 933 | **α-pinene** | 1.9 |
| 13.5 | 1027 | 1030 | **limonene** | 1.9 |
| 11.2 | 974 | 978 | **β-pinene** | 1.7 |
| ***Syzygium aromaticum*** | | | | |
| *RetT* | *RI (Ms)* | *RI (Db)* | *Compound* | *%* |
| 28.6 | 1350 | 1357 | **eugenol** | 85.1 |
| 35.3 | 1510 | 1521 | eugenyl acetate | 7.3 |
| 31.4 | 1415 | 1424 | ***trans*-caryophyllene** | 5.4 |
| ***Evernia prunastri**** | | | | |
| *RetT* | *RI (Ms)* | *RI (Db)* | *Compound* | *%* |
| 42.5 | 1696 | *1708* | **methyl atrarate** | 100.0 |
| ***Pimenta dioica*** | | | | |
| *RetT* | *RI (Ms)* | *RI (Db)* | *Compound* | *%* |
| 28.5 | 1350 | *1357* | **eugenol** | 71.6 |
| 30.6 | 1396 | 1403 | methyl eugenol | 12.5 |
| 31.4 | 1415 | 1424 | ***trans*-caryophyllene** | 8.0 |
| 32.8 | 1450 | 1454 | α-humulene | 1.7 |
| ***Pelargonium graveolens*** | | | | |
| *RetT* | *RI (Ms)* | *RI (Db)* | *Compound* | *%* |
| 23.0 | 1227 | *1232* | citronellol | 35.7 |
| 24.0 | 1250 | 1255 | geraniol | 14.8 |
| 17.4 | 1109 | 1113 | phenethyl alcohol | 10.4 |
| 29.7 | 1377 | 1380 | ***trans*-geranyl acetate** | 7.1 |
| 25.1 | 1272 | 1275 | citronellyl formate | 4.6 |
| 28.9 | 1357 | 1361 | *cis*-geranyl acetate | 3.6 |
| 22.8 | 1223 | 1228 | nerol | 2.9 |
| 16.9 | 1099 | 1101 | **linalool** | 2.8 |
| 19.9 | 1162 | 1166 | isomenthone | 2.8 |
| 39.5 | 1617 | 1632 | δ-eudesmol | 2.7 |
| 26.2 | 1297 | 1284 | lavandulyl acetate | 1.4 |
| 28.5 | 1349 | 1350 | citronellyl acetate | 1.1 |
| ***Bulnesia sarmientoi*** | | | | |
| *RetT* | *RI (Ms)* | *RI (Db)* | *Compound* | *%* |
| 41.2 | 1660 | 1673 | bulnesol | 40.3 |
| 38.6 | 1592 | 1603 | guaiol | 34.9 |
| 40.7 | 1648 | 1656 | β-eudesmol | 7.5 |
| 39.9 | 1626 | 1632 | δ-eudesmol | 3.2 |
| 39.5 | 1616 | 1620 | eudesmol (10-epi-δ) | 1.8 |
| 39.0 | 1603 | 1614 | guaiol | 1.6 |
| 39.1 | 1606 | 1609 | rosifoliol | 1.2 |
| ***Thymus hiemalis*** | | | | |
| *RetT* | *RI (Ms)* | *RI (Db)* | *Compound* | *%* |
| 13.6 | 1029 | 1032 | **eucalyptol** | 30.6 |
| 19.0 | 1142 | 1149 | camphor | 9.3 |
| 16.9 | 1098 | 1101 | **linalool** | 8.6 |
| 10.0 | 945 | 942 | camphene | 5.9 |
| 13.3 | 1022 | 1025 | ***p*-cymene** | 4.8 |
| 20.2 | 1168 | 1173 | borneol | 4.6 |
| 9.3 | 930 | 933 | **α-pinene** | 3.7 |
| 14.4 | 1044 | 1046 | *trans*-β-ocimene | 3.1 |
| 11.8 | 987 | 991 | myrcene | 3.0 |
| 21.3 | 1192 | 1195 | **α-terpineol** | 2.6 |
| 11.2 | 973 | 978 | **β-pinene** | 2.4 |
| 31.4 | 1415 | 1424 | ***trans*-caryophyllene** | 2.1 |
| 24.7 | 1265 | 1268 | geranial | 2.0 |
| 14.9 | 1055 | 1058 | δ-terpinene | 1.9 |
| 13.5 | 1026 | 1030 | **limonene** | 1.8 |
| 20.6 | 1177 | 1184 | terpinen-4-ol | 1.8 |
| 11.0 | 969 | 972 | sabinene | 1.4 |
| 23.4 | 1235 | 1238 | neral | 1.4 |
| 34.5 | 1491 | 1497 | bicyclogemacrene | 1.1 |
| ***Canarium luzonicum*** | | | | |
| *RetT* | *RI (Ms)* | *RI (Db)* | *Compound* | *%* |
| 13.5 | 1027 | 1030 | **limonene** | 50.1 |
| 36.6 | 1544 | 1546 | α-elemol | 25.7 |
| 12.4 | 1003 | 1007 | α-phellandrene | 6.7 |
| 11.0 | 969 | 972 | sabinene | 4.7 |
| 13.3 | 1021 | 1025 | ***p*-cymene** | 3.2 |
| 21.3 | 1191 | 1195 | **α-terpineol** | 2.4 |
| ***Dalbergia sissoo*** | | | | |
| *RetT* | *RI (Ms)* | *RI (Db)* | *Compound* | *%* |
| 16.9 | 1099 | *1101* | **linalool** | 79.8 |
| 21.3 | 1191 | 1195 | **α-terpineol** | 6.1 |
| 13.6 | 1029 | 1032 | **eucalyptol** | 3.8 |
| 16.3 | 1085 | - | NI: 71(100),43(38),68(28),41(22) | 3.6 |
| 23.9 | 1248 | 1244 | geraniol | 2.7 |
| 13.5 | 1026 | 1030 | **limonene** | 1.7 |
| ***Illicum verum*** | | | | |
| *RetT* | *RI (Ms)* | *RI (Db)* | *Compound* | *%* |
| 25.6 | 1284 | 1288 | *trans*-anethole | 90.92 |
| 21.5 | 1194 | 1201 | estragole | 3.10 |
| 13.5 | 1026 | 1030 | **limonene** | 1.22 |
| ***Cymbopogon martinii*** | | | | |
| *RetT* | *RI (Ms)* | *RI (Db)* | *Compound* | *%* |
| 24.1 | 1251 | 1255 | geraniol | 76.87 |
| 29.7 | 1375 | 1380 | ***trans*-geranyl acetate** | 9.63 |
| 31.3 | 1414 | 1424 | ***trans*-caryophyllene** | 3.54 |
| 16.9 | 1098 | 1101 | **linalool** | 2.80 |
| 13.5 | 1026 | 1030 | **limonene** | 1.22 |
| ***Agathosma betulina*** | | | | |
| *RetT* | *RI (Ms)* | *RI (Db)* | *Compound* | *%* |
| 19.5 | 1153 | 1158 | menthone | 32.5 |
| 13.5 | 1026 | 1030 | **limonene** | 20.0 |
| 19.9 | 1161 | 1166 | isomenthone | 11.5 |
| 26.0 | 1293 | 1299 | diosphenol | 8.5 |
| 24.5 | 1261 | 1267 | pseudodiosphenol | 7.4 |
| 23.3 | 1234 | 1241 | pulegone | 5.3 |
| 13.7 | 1029 | 1032 | **eucalyptol** | 3.8 |
| 9.3 | 930 | 933 | **α-pinene** | 2.5 |
| 11.8 | 988 | 991 | **myrcene** | 1.4 |
| 24.0 | 1248 | 1258 | NI: 43(100),69(92),112(92),70(52),55(49) | 1.2 |
| 20.7 | 1177 | 1184 | **terpinen-4-ol** | 1.1 |
| ***Citrus bergamia*** | | | | |
| *RetT* | *RI (Ms)* | *RI (Db)* | *Compound* | *%* |
| 13.5 | 1027 | 1030 | **limonene** | 37.0 |
| 24.0 | 1249 | 1250 | linalyl acetate | 31.1 |
| 16.9 | 1098 | 1101 | **linalool** | 11.3 |
| 11.2 | 974 | 978 | **β-pinene** | 6.7 |
| 14.9 | 1056 | 1058 | γ-terpinene | 6.6 |
| 9.3 | 930 | 933 | **α-pinene** | 1.1 |
| ***Salvia sclarea*** | | | | |
| *RetT* | *RI (Ms)* | *RI (Db)* | *Compound* | *%* |
| 24.0 | 1249 | 1250 | linalyl acetate | 62.2 |
| 16.9 | 1098 | 1101 | **linalool** | 23.3 |
| 21.3 | 1192 | 1195 | **α-terpineol** | 4.5 |
| 29.7 | 1375 | 1380 | ***trans*-geranyl acetate** | 2.4 |
| 31.3 | 1414 | 1424 | ***trans*-caryophyllene** | 1.5 |
| 28.8 | 1356 | 1361 | *cis*-geranyl acetate | 1.2 |
| 33.9 | 1476 | 1478 | **γ-muurolene** | 1.2 |
| ***Ravensara aromatica*** | | | | |
| *RetT* | *RI (Ms)* | *RI (Db)* | *Compound* | *%* |
| 13.5 | 1026 | 1030 | **limonene** | 16.0 |
| 11.0 | 969 | 972 | sabinene | 9.8 |
| 30.6 | 1396 | 1403 | methyl eugenol | 8.2 |
| 31.3 | 1414 | 1424 | ***trans*-caryophyllene** | 6.1 |
| 13.3 | 1021 | 1025 | ***p*-cymene** | 5.3 |
| 16.9 | 1097 | 1101 | **linalool** | 5.0 |
| 33.9 | 1476 | 1478 | γ-muurolene | 4.8 |
| 9.3 | 929 | 933 | **α-pinene** | 4.4 |
| 20.6 | 1176 | 1184 | **terpinen-4-ol** | 3.9 |
| 36.6 | 1543 | 1551 | elemicin | 3.3 |
| 21.4 | 1194 | 1201 | estragole | 3.3 |
| 12.5 | 1006 | 1009 | δ-3-carene | 3.1 |
| 11.1 | 973 | 978 | **β-pinene** | 2.7 |
| 12.9 | 1014 | 1018 | α-terpinene | 2.3 |
| 11.7 | 987 | 991 | **myrcene** | 1.8 |
| 13.6 | 1028 | 1032 | **eucalyptol** | 1.5 |
| 10.0 | 945 | 953 | camphene | 1.4 |
| 32.8 | 1450 | 1454 | α-humulene | 1.3 |
| 29.5 | 1371 | 1375 | α-copaene | 1.2 |
| ***Matricaria chamomilla*** | | | | |
| *RetT* | *RI (Ms)* | *RI (Db)* | *Compound* | *%* |
| 14.6 | 1048 | 1050 | isobutyl angelate | 20.9 |
| 32.9 | 1451 | 1452 | *trans*-β-farnesene | 15.7 |
| 44.1 | 1742 | 1748 | α-bisabolol oxide a | 15.5 |
| 19.3 | 1150 | 1151 | 2-methylbutyl angelate | 11.5 |
| 15.2 | 1062 | 1072 | (2z)-hexenyl tiglate | 5.3 |
| 40.7 | 1649 | 1655 | α-bisabolol oxide b | 3.5 |
| 41.7 | 1675 | 1682 | α-bisabolone oxide a | 3.4 |
| 18.7 | 1137 | 1141 | trans-pinocarveol | 3.3 |
| 19.1 | 1146 | 1149 | isoamyl angelate | 2.9 |
| 8.6 | 912 | 913 | isobutyl isobutyrate | 2.0 |
| 13.0 | 1014 | 1014 | isopentyl isobutyrate | 1.3 |
| 33.9 | 1476 | 1478 | **γ-muurolene** | 1.2 |
| ***Cananga odorata*** | | | | |
| *RetT* | *RI (Ms)* | *RI (Db)* | *Compound* | *%* |
| 33.9 | 1476 | 1478 | **γ-muurolene** | 18.9 |
| 19.8 | 1159 | 1167 | benzyl acetate | 11.9 |
| 35.0 | 1501 | 1504 | (*trans*,*trans*)-α-farnesene | 8.8 |
| 16.9 | 1098 | 1101 | **linalool** | 8.1 |
| 44.7 | 1758 | 1772 | benzyl benzoate | 7.2 |
| 13.1 | 1017 | 1022 | *p*-methyl anisole | 6.6 |
| 31.4 | 1415 | 1424 | ***trans*-caryophyllene** | 5.0 |
| 29.7 | 1376 | 1380 | ***trans-*geranyl acetate** | 4.5 |
| 16.5 | 1091 | 1096 | clorius | 4.2 |
| 32.4 | 1440 | 1448 | *trans*-cinnamyl acetate | 4.0 |
| 47.2 | 1828 | 1832 | farnesyl acetate | 2.8 |
| 48.3 | 1860 | 1872 | benzyl salicylate | 2.3 |
| 32.9 | 1451 | 1454 | α-humulene | 1.9 |
| 35.5 | 1514 | 1518 | δ-cadinene | 1.8 |
| 40.7 | 1649 | 1659 | cadin-4-en-10-ol | 1.5 |
| 8.9 | 919 | 920 | prenyl acetate | 1.1 |
| ***Cedrus atlantica*** | | | | |
| *RetT* | *RI (Ms)* | *RI (Db)* | *Compound* | *%* |
| 34.8 | 1496 | 1503 | β-himachalene | 47.2 |
| 32.6 | 1445 | 1449 | α-himachalene | 17.2 |
| 33.8 | 1474 | 1482 | γ-himachalene | 10.5 |
| 45.1 | 1767 | 1775 | *trans*-α-atlantone | 2.6 |
| 34.0 | 1478 | - | himachalene-1,4-diene | 2.1 |
| 35.5 | 1514 | 1518 | δ-cadinene | 1.8 |
| 35.7 | 1520 | 1530 | γ-dehydro-ar-himachalene | 1.6 |
| 39.1 | 1607 | 1618 | β-himachalene oxide | 1.4 |
| 36.4 | 1536/ 1537 | 1544/ 1540 | *trans*-α-bisabolene/  α-calacorene | 1.2 |
| ***Artemisia pallens*** | | | | |
| *RetT* | *RI (Ms)* | *RI (Db)* | *Compound* | *%* |
| 37.9 | 1576 | 1582 | davanone D | 56.4 |
| 34.6 | 1492 | 1497 | bicyclogermacrene | 8.5 |
| 33.4 | 1463 | 1473 | *trans*-ethyl-cinnamate | 4.1 |
| 34.9 | - | - | NI: 109(100),43(72),124(59) | 3.1 |
| 34.3 | 1485 | 1492 | β-selinene | 1.6 |
| 35.7 | - | - | NI: 109(100),43(70),124(57) | 1.6 |
| 40.3 | 1638 | 1640 | epi-α-cadinol | 1.6 |
| 37.8 | 1572 | 1576 | spathulenol | 1.6 |
| 37.1 | 1554 | 1557 | davanone B | 1.5 |
| 29.5 | 1372 | - | *cis*-ethyl-cinnamate | 1.2 |
| ***Cyperus scariosus*** | | | | |
| *RetT* | *RI (Ms)* | *RI (Db)* | *Compound* | *%* |
| 30.6 | 1398 | 1407 | cyperene | 26.3 |
| 33.1 | 1457 | 1456 | rotundene | 7.6 |
| 42.2 | 1688 | 1693 | cyperotundone | 5.6 |
| 29.5 | 1372 | 1375 | α-copaene | 4.8 |
| 34.4 | 1488 | 1491 | eremophilene | 3.8 |
| 37.9 | 1576 | 1567 | caryophyllene oxide | 3.0 |
| 34.3 | 1484 | 1492 | β-selinene | 2.7 |
| 41.5 | 1667 | 1681 | mustakone | 2.5 |
| 43.9 | - | - | NI:93(100),91(83),218(86),  107(72) | 2.3 |
| 35.5 | 1514 | 1518 | selinene <7-epi-alpha-> | 2.2 |
| 39.5 | - | - | NI: 218(100),175(95),147(51) | 2.2 |
| 33.6 | 1468 | 1476 | γ-gurjunene | 1.9 |
| 36.0 | 1528 | - | cyperene epoxide | 1.5 |
| 34.5 | 1491 | 1501 | α-selinene | 1.4 |
| 34.8 | 1497 | 1505 | α-bulnesene | 1.4 |
| 34.1 | 1479 | 1490 | aristochelene | 1.4 |
| 29.0 | 1360 | - | *trans*-valerenyl acetate | 1.3 |
| 40.8 | - | - | NI:91(100),93(99),105(83)107(83)157(81) | 1.3 |
| 11.2 | 975 | 978 | **β-pinene** | 1.1 |
| 42.4 | - | - | NI: 123(100),95(79),81(78) | 1.1 |
| 32.6 | 1445 | 1438 | α-guaiene | 1.0 |
